# Early risk assessment of circulating endothelial progenitor cells and plasma stromal cell-derived factor-1 for nondisabling ischemic cerebrovascular events

**DOI:** 10.1186/s12883-019-1250-5

**Published:** 2019-02-12

**Authors:** Wang Zhao, Libo Zhao, Juan Liao, Yong Luo, Lanying He

**Affiliations:** 1 Department of Neurology, Yongchuan Hospital of Chongqing Medical University, Chongqing, 402160 China; 2 Central Laboratory, Chongqing Key Laboratory of Cerebrovascular Disease Research, Yongchuan Hospital of Chongqing Medical University, Chongqing, 402160 China; 3 Department of Neurology, The First Affiliated Hospital of Chongqing Medical University, Chongqing, 400016 China; 4 Department of Neurology, Second Peoples Hospital of Chengdu, Chengdu, 610000 Sichuan Province China

**Keywords:** Endothelial progenitor cells, Stromal cell-derived factor-1, TIA;minor stroke, Nondisabling ischemic cerebrovascular events

## Abstract

**Background:**

Endothelial progenitor cells (EPCs) play an important role in ischemic stroke. However, there are few studies on the relationship between EPC and nondisabling ischemic cerebrovascular events. Our aim was to investigate the association of EPCs and SDF-1 (serum stromal cell-derived factor-1) with NICE (nondisabling ischemic cerebrovascular events).

**Methods:**

TIA (transient ischemic attack) and minor stroke patients (153 in total) who had an onset of symptoms within 1 day were consecutively collected. 83 of the patients were categorized into the HR-NICE (high-risk nondisabling ischemic cerebrovascular event) group, and 70 of the patients were in the NHR-NICE (non-high-risk nondisabling ischemic cerebrovascular events) group. Adopted FCM (flow cytometry) was used to measure EPCs, taking double-positive CD34/KDR as EPCs. ELISA was used to measure the concentrations of serum SDF-1 and VEGF (vascular endothelial growth factor). By the sequence of admission time, 15 patients were selected separately from the HR-NICE group and NHR-NICE group, and another 15 healthy volunteers were chosen as the NC (Normal Control) group. The MTT method was used to measure the proliferation of EPCs of peripheral blood in all groups, and the Boyden chamber was used to measure the migration of EPCs.

**Results:**

Compared with the NHR-NICE group, the HR-NICE group was older and contained more patients with hypertension and diabetes. Triglyceride, total cholesterol, and low-density lipoprotein in the HR-NICE group were also higher. For factors such as smoking, BMI (body mass index), and HCY (homocysteine), there were no significant differences (*P* > 0.05). Circulating EPCs, SDF-1, and VEGF in the NHR-NICE group were all higher. According to the multifactor regression analysis, age, hypertension, diabetes, total cholesterol, EPCs, and SDF-1 are independent risk factors for HR-NICE. For EPCs of 48-h isolated cultures, proliferation and migration were observed to be weakened compared with those of the NC group (*P* < 0.05). EPCs in HR-NICE group had lower proliferation and migration than those in NHR-NICE group (*P* < 0.01).

**Conclusions:**

For TIA and minor stroke patients, circulating EPCs and serum SDF-1 concentrations can be used to prognose HR-NICE. Factors that lead to high-risk NICE might be relevant to the decrease in proliferation and migration of circulating EPCs.

## Background

Endothelial progenitor cells (EPCs) play an important role in tissue repair in ischemic organs. Many studies have been conducted to demonstrate that endothelial progenitor cells promote ischemic tissue angiogenesis after stroke. However, there are few studies on the relationship between EPC and nondisabling ischemic cerebrovascular events. According to CNSR II (The China National Stroke Registry II), 23,900,000 patients suffer from TIA [1], and minor stroke patients account for 46.4% of all ischemic stroke patients. We should treat TIA and minor stroke as the most important group for prevention and treatment. The imbalance of the amount of vessel damage and the abilities of the repair system can lead to atherosclerosis and then cerebral infarction. Regarding vessel repair, previous studies have shown that plasma SDF-1 has a positive impact on the long-term prognosis of ACI (acute cerebral infarction) [2]. After ACI, plasma SDF-1 will increase, and a large amount of EPCs in peripheral blood will participate in the regeneration and repair of vessels [3]. When EPCs were found to be increased in patients who suffered TIA for the first time, the possibility of experiencing an ischemic attack in the future wasn’t increased [4]. Based on previous studies, we are enlightened that differences in the quantity and quality of circulating EPCs as well as serum SDF-1 levels might exist in patients with TIA and minor stroke. These differences might be used to differentiate NHR-NICE and HR-NICE, where HR-NICE may develop into DICE (disabling ischemic cerebrovascular events). Therefore, circulating EPCs and SDF-1 levels might be able to further prognose high-risk cerebral stroke for NICE patients. China is one of the countries that suffers most from cerebral stroke [5], with an enormous amount of HR-NICE patients; therefore, it is very beneficial to study the relationship between EPCs, SDF-1 and NICE.

## Methods

### Research subjects

A total of 186 TIA and minor stroke patients were consecutively collected from June 2016 to June 2018 in the Neurology Department of the Affiliated Hospital of Chongqing Medical University, from both out-patient service and hospitalization. All patients developed morbidity within 1 day. Their ages ranged from 18 to 80 years; 12 were excluded cases, and 21 were shedding cases. The NC group consisted of 15 healthy volunteers who were openly recruited after an official announcement from the hospital during the same time period. Their genders and ages were matched to the other two groups. Finally, 153 patients and 15 healthy volunteers were included in the analysis. Among these 153 patients, there were 83 HR-NICE patients and 70 NHR-NICE patients. Blood samples were taken immediately after admission for TIA and minor stroke patients, and FCM was adopted to measure the amount of circulating EPCs. Using ELISA, the concentrations of serum SDF-1 and VEGF were measured. For the 15 patients in the NC group, only circulating EPCs were extracted. Meanwhile, circulating EPCs were extracted from the first 15 patients in both the HR-NICE and NHR-NICE groups. The MTT method was used to measure the proliferation of EPCs of peripheral blood in all groups, and the Boyden chamber was used to measure the migration of EPCs. NICE refers to ischemic cerebrovascular events that leave no significant disability after morbidity, which includes three types of patients: 1. those who had a TIA; 2. those who had a minor stroke; and 3. those whose symptoms rapidly alleviated and left no significant disability after morbidity. In this research, the definition of HR-NICE is as follows: high-risk TIA (ABCD2 score ≥ 4 points) and minor stroke patients who developed morbidity within 24 h [1]. The definition of NHR-NICE is TIA (ABCD2 score < 4 points). Participants were excluded if they had any history of the following: trauma, blood system diseases, renal insufficiency, inflammation, ulcer, abnormal liver function, malignant tumors, myocardial infarction, angina, atrial fibrillation, recent surgical history, and usage of statins or EPO within 2 months before morbidity. Minor stroke can be defined as NIHSS score ≤ 3; The definition of “no significant disability after morbidity” is as follows: severe symptoms during morbidity but the symptoms alleviated as TIA or minor stroke as in treatment. This research was approved by the Ethics Committee of Yongchuan Hospital of Chongqing Medical University. All patients or their relatives signed an informed consent form.

### Measure of circulating EPCs

CD34+/KDR+ were adopted as a marker of circulating EPCs [6]. After admission, 8 mL of antecubital venous blood was extracted and added to a buffer solution with 3.8% heparin. A density gradient centrifugation was used to obtain a monocyte layer and to adjust the density of the cells to 1 × 10^6^/ml. 20 μl of Mouse Anti-Human CD34 monoclonal IgG1 (Southern Biotech Inc., U.S.) was added with a PE mark and 10 μl of Mouse Anti-Human KDR monoclonal IgG1 (R&D Inc., U.S.) was added with an FITC mark. These were then incubated at room temperature without light for 30 min. Next, the samples were centrifuged at 4 °C for 5 min at 1500 rpm/min, and the supernatant was removed and washed twice with PBS. Then, FCM was used to conduct quantitative analysis. The percentage of CD34+/KDR+ cells account for monocyte cells and were used to measure the level of EPCs.

### Identification of EPCs

Cultivated monocytes were obtained by density gradient centrifugation in M199 medium (Hyclone, USA) until cell climbing on the 7th day. These were fixed with 2% paraformaldehyde for 15 min and washed in PBS 3 times. Fifty microliters of DIL-acLDL (10 μg/ml) was added then the cells were cultivated at 37 °C in an incubator for 1 h. Next, they were washed twice with PBS, and fixed with 2% paraformaldehyde for 10 min at room temperature. Fifty microliters of FITC-UEA-Lectin (20 μg/ml) was added, and then the cells were cultivated for 2 h at 4 °C without light and washed with PBS three times. The cells were identified by laser confocal microscopy, where FITC-Lectin positive was in green fluorescence, DIL-acLDL positive was in red fluorescence, and the two target cells were yellow, which were the EPCs under differentiation.

### Proliferation of EPCs

The 3-(4,5-dimethylthiazol-2-yl)-2,5-diphenyltetrazolium bromide (MTT) assay was performed according to the protocol of the manufacturer to determine the proliferation of EPCs [7]. The density of cultured EPCs was adjusted for 48 h to 1 × 10^6^/ml, and 100 μl of EPCs were inoculated into the culture plate coated with human fibronectin. Then, 20 μl of MTT solution (5 mg/ml) was added to each hole and cultivated for 6 h. The supernatant was removed. Then, 150 μl of dimethyl sulfoxide was added to each hole and oscillated for 10 min. The absorbance was measured at 490 nm by automatic enzyme labeling. For the clonogenic assay, the cells were seeded in 60 mm dishes (1000 cells/plate). Twelve hours after seeding, three groups of cells were allowed to grow until visible colonies appeared. The colonies were stained with 0.01% crystal violet (Sigma, St. Louis, MO) and counted under a microscope.

### Migration of EPCs

Cell migration was quantified using a Boyden chamber. Briefly, the density of the EPCs cultured for 48 h was adjusted to 1 × 10^6^/ml. A 50 μl cell suspension was added to the upper chamber of the Boyden chamber. Then, 25 μl of culture medium and VEGF (50 ng/ml) were added to the lower chamber of the Boyden chamber. Cells were cultivated in an incubator for 6 h. Then, the cells were stained at high magnification. Immobile cells were scraped off the membrane, fixed with methanol, Giemsa stained, and counted under a high-power lens.

### Measure concentrations of serum SDF-1 and VEGF

Three milliliters of antecubital venous blood was extracted and added to a buffer solution with 3.8% heparin. Then, the solution was centrifuged at 4 °C for 15 min at 3000 rpm/min, and the supernatant was collected and kept it in a − 80 °C freezer. ELISA was performed according to the protocol of the manufacturer to determine the concentration of serum SDF-1 and VEGF.

### Statistical analysis

The SPSS version 21.0 statistics software was used for analysis. The continuous variable is expressed as the mean ± standard deviation, and the categorical variables are expressed as percentages. Chi-square test, T test and ANOVA (analysis of variance) were used to compare the differences among the groups. The relationship between risk factors for traditional cerebrovascular diseases, EPCs, SDF-1, VEGF and NICE was evaluated by logistic regression analysis. *P* < 0.05 is taken to show statistical significance.

## Results

### Baseline characteristics

The comparison of clinical data between the NHR-NICE and HR-NICE groups is in Table 1. There were 85 males and 68 females in the 153 TIA and minor stroke cases, whose average age was 59.15 years old. However, patients in the HR-NICE group were elderly and had more hypertension and diabetes (*P* < 0.01). The laboratory data showed that compared with the NHR-NICE group, patients in HR-NICE had higher levels of peripheral blood triglyceride, total cholesterol, and low-density lipoprotein. Regarding the other variables such as the sampling time, gender, smoking, BMI (Body Mass Index), and HCY (homocysteine), there were no significant differences between the two groups (*P* > 0.05).

**Table 1:** Clinical and laboratory data of NHR-NICE and HR-NICE group

	NHR-NICE(70)	HR-NICE(83)	P
Time to sampling(h)	10.69 ± 5.00	9.98 ± 4.69	0.370
Age (year)	56.50 ± 8.16	62.63 ± 5.29	<0.01
Male n (%)	40(57.14%)	45(54.22%)	0.719
Hypertension n (%)	15(21.43%)	46(55.42%)	<0.01
Diabetes n (%)	7(10.00%)	33(39.76%)	<0.01
Smoking n (%)	28(40.00%)	24(28.92%)	0.149
BMI≧24 n (%)	27(38.57%)	36(43.37)	0.548
TG (mmol/L)	2.14 ± 0.59	2.44 ± 0.59	<0.01
TC (mmol/L)	5.32 ± 0.74	5.67 ± 0.74	<0.01
LDL (mmol/L)	3.42 ± 0.60	3.64 ± 0.70	0.018
HCY umol/L	14.48 ± 4.70	14.30 ± 5.79	0.514
EPCs(EPCs/PMNC,‱)	5.75 ± 0.97	4.09 ± 0.77	<0.01
SDF-1 (pg/ml)	470.30 ± 20.14	755.12 ± 29.67	<0.01
VEGF (pg/ml)	70.97 ± 3.27	83.28 ± 2.36	<0.01

Time to sampling: time from onset to collect blood sample;*BMI* Body Mass Index, *TG* Triglyceride, *TC* Total Cholesterol, *LDL* Low Density Lipoprotein, *EPCs* Endothelial Progenitor Cells, *SDF-1* Stromal Cell-Derived Factor-1, *VEGF*, Vascular Endothelial Growth Factor

### Expression of EPCs, SDF-1 and VEGF in the NHR-NICE group and HR-NICE group

**Fig. 1:**
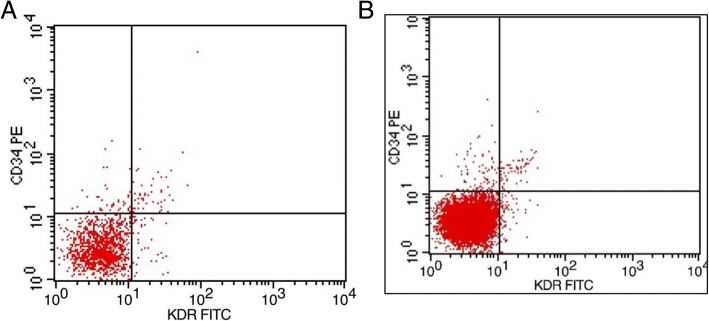
Flow cytometry results revealed double-positive CD34/KDR cells in each group: in the HR-NICE group (Fig. 1a) and the NHR-NICE group (Fig. 1b)

The expression of EPCs, SDF-1 and VEGF in the NHR-NICE group and HR-NICE group is shown in Table 1 above. The data show that the circulating EPCs in the NHR-NICE group accounted for 5.75/000 of monocytes (Fig. 1a), while those in the HR-NICE group accounted for 4.09/000 (Fig. 1b). The mean values of serum SDF-1 in the two groups were 470.30 pg/ml and 755.12 pg/ml and inter-assay CV of 4.1%, respectively. The mean serum VEGF values of the two groups were 70.97 pg/ml and 83.28 pg/ml and inter-assay CV of 3.7%, respectively. In addition, the intra-assay CV of serum SDF in the NHR-NICE group and HR-NICE group were 4.3 and 3.9%, the intra-assay CV of serum VEGF in the two groups were 4.6 and 2.8%. The difference between the two groups is significant (*P* < 0.01).

### Analysis of related risk factors of NICE

Univariate logistic regression analysis screened for possible variables, and the results showed that age, hypertension, diabetes, triglycerides, total cholesterol, low-density lipoprotein, EPCs, SDF-1, VEGF were associated with HR-NICE. However, the risk factors for traditional atherosclerosis include obesity, smoking, and homocysteine, so BMI, smoking, and homocysteine are also included in the multivariate regression analysis. After adjusting for age, BMI, hypertension, diabetes, smoking, triglycerides, total cholesterol, low-density lipoprotein, homocysteine, EPCs, SDF-1, and VEGF for multivariate logistic regression analysis, the results showed the following: age (OR:1.134, 95% CI: 1.030–1.249, *P* = 0.011), hypertension (OR: 10.798, 95% CI: 2.174–53.622, *P* = 0.004), diabetes (OR: 11.630, 95% CI: 1.487–90.941, *P* = 0.019), TC (OR: 2.416, 95% CI: 0.971–6.010, *P* = 0.058), EPCs (OR: 0.067, 95% CI: 0.023–0.198, *P* < 0.01), and SDF-1 (OR: 1.007, 95% CI: 1.003–1.011, P < 0.01) are all independent risk factors for HR-NICE (Table 2).

**Table 2:** Analysis of related risk factors of HR-NICE

Variables	P	OR	95%CI
Univariate analysis			
Age	<0.01	1.145	1.075–1.218
Gender	0.717	1.126	0.593–2.137
IBM	0.548	1.220	0.638–2.332
Hypertension	<0.01	0.219	0.107–0.449
Diabetes	<0.01	5.940	2.424–14.553
Smoking	0.151	0.610	0.311–1.197
TG	0.002	2.517	1.384–4.578
TC	0.005	1.897	1.214–2.966
LDL	0.040	1.723	1.024–2.899
Homocysteine	0.836	0.994	0.936–1.055
EPCs	<0.01	0.123	0.065–0.233
SDF-1	<0.01	1.005	1.003–1.007
VEGF	<0.01	1.016	1.004–1.029
Multivariate analysis			
Age	.011	1.134	1.030–1.249
Hypertension	.004	10.798	2.174–53.622
Diabetes	.019	11.630	1.487–90.941
TC	.058	2.416	0.971–6.010
EPCs	<0.01	0.067	0.023–0.198
SDF-1	.001	1.007	1.003–1.011

### Identification, proliferation and migration of EPCs

**Fig. 2:**
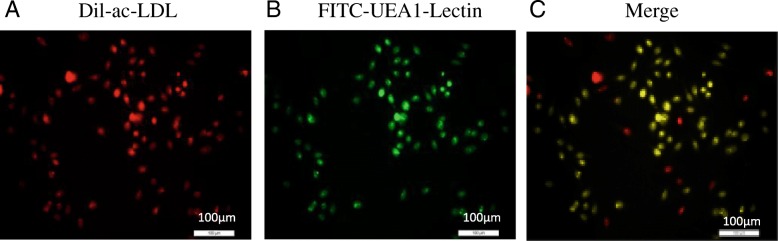
EPC double phagocytosis experiments showed that FITC-Lectin was positive for green fluorescence (Fig. 2a), DIL-acLDL was positive for red fluorescence (Fig. 2b), and double-labeled cells were yellow for the differentiation of EPCs (Fig. 2c)

**Fig. 3:**
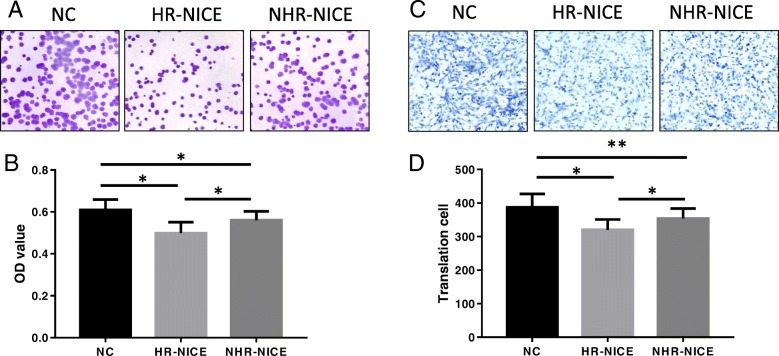
Peripheral blood was isolated and cultured for 48 h in EPCs. A colony assay (Fig. 3a, magnification × 200) and MTT assay (Fig. 3b) were used to detect the proliferation ability of each group. The Transwell assay was used to test the cell migration ability (Fig. 3c, magnification × 100); and the counting chart is shown in Fig. 3d. The cell migration ability of the HR-NICE group was weakened (*P* < 0.01). The cell migration ability of the NHR-NICE group was weaker than that of the NC group, and the difference was significant (*P* < 0.05). *P < 0.01, **P < 0.05

EPC double phagocytosis experiments showed that FITC-Lectin was positive when green fluorescence was present (Fig. 2a), DIL-acLDL was positive when red fluorescence was present (Fig. 2b), and double-labeled cells were yellow for the differentiation of EPCs (Fig. 2c). Peripheral blood was isolated and cultured for 48 h in EPCs. An MTT assay was used to detect the proliferation and migration ability of each group. The results showed that compared with the NC group, the cell proliferation abilities of the HR-NICE group and the NHR-NICE group were inhibited, and the difference was very significant (*P* < 0.01, Fig. 3a, b). The cell migration ability of the HR-NICE group was weakened (P < 0.01, Fig. 3c, d). The cell migration ability of the NHR-NICE group was weaker than that of the NC group, and the difference was significant (*P* < 0.05). Compared with the NHR-NICE group, the cell proliferation and migration ability of the HR-NICE group was decreased (*P* < 0.01).

## Discussion

The regeneration and repair of blood vessels after ischemic stroke have been the subjects of many studies. In 2010, the concept of minor stroke was proposed, and a stroke with an NIHSS score of less than 3 was classified as minor stroke [8].

In 2016, China published the “Guidelines for the diagnosis and treatment of high-risk nondisabling ischemic cerebrovascular events”. Most of the nondisabling ischemic cerebrovascular events were TIA and minor stroke. At present, a variety of factors can predict the risk of early stroke recurrence in NICE, mainly divided into clinical predictors [9–12], imaging predictors [13–15] and biomarker predictors [16–18], in which clinical predictors have been studied the most abundantly but not a single biomarker predictor has been found for clinical application.

Studies have shown that approximately 5 to 25% of the endothelium in neovascularization after cerebral infarction is derived from EPCs [19]. EPCs are considered to be immature endothelial cells that can promote angiogenesis. The most widely accepted phenotypically defined EPC is the coexpression of the cell-surface markers CD34 and VEGFR2 [20]. Some studies have also suggested that low circulating EPC levels were independently predictive of severe neurological impairment (National Institutes of Health Stroke Scale ≥12) at 48 h and of combined major adverse clinical outcomes on day 90 after ischemic stroke [21].

Studies from Ghani showed that patients with fewer EPCs in their peripheral blood are more likely to develop cerebrovascular disease. They proposed that the number of peripheral blood EPCs can be used as an indicator of vascular repair function and an independent predictor of cardiovascular and cerebrovascular events [22]. Some scholars have suggested that circulating EPCs and SDF-1 levels may predict the severity and prognosis of patients with ischemic stroke. Higher levels of EPCs in peripheral blood indicate a smaller infarct volume in stroke and a slower development rate. Compared with cerebral infarction of small blood vessels, the level of circulating EPCs in patients with cerebral infarction with large vascular disease is low, and the lower level of circulating EPCs at admission indicates a poor prognosis within 6 months [23–26]. Regarding the study of EPCs, previous studies have been conducted on patients with cerebral infarction and myocardial infarction, and there are few studies on patients with TIA and minor stroke. Scholars still pay little attention to the application of EPCs in patients with nondisabling ischemic cerebrovascular events.

This research is based on 153 patients with TIA and minor stroke. The data showed that patients in the HR-NICE group were older and included more patients with hypertension and diabetes. The reason for this is related to whether the patients were classified as high-risk nondisabling ischemic cerebrovascular events using the ABCD2 scoring criteria because age, hypertension, and diabetes were some of the indicators of the ABCD2 score. However, peripheral blood triglycerides, total cholesterol, and low-density lipoprotein were also higher in the HR-NICE group and more risk factors for stroke were found in this group as well.

At 24 h after onset, the HR-NICE group had lower circulating EPC levels and higher serum SDF-1 and VEGF levels, which may be more related to the risk factors of this group of patients and is consistent with reports that showed more risk factors with lower EPC levels [27, 28]. Studies have reported that an increased probability of future cerebrovascular events did not occur in these patients with elevated EPCs in the first attack of TIA [4], which is consistent with higher levels of EPCs in the NHR-NICE group in this study. In other words, the reduction in EPCs may increase the risk of recurrent stroke in NICE patients. After adjusting for age, BMI, hypertension, diabetes, smoking, triglycerides, total cholesterol, low-density lipoprotein, homocysteine, EPCs, SDF-1, and VEGF, multivariate logistic regression analysis showed that age, hypertension, diabetes, EPCs, and SDF-1 are independent risk factors for HR-NICE. The results of the proliferation and migration of EPCs suggested that the proliferation and migration abilities of the HR-NICE group and NHR-NICE group were weaker than those of the NC group and that those of the HR-NICE group was worse. Compared with the NHR-NICE group, the number of circulating EPCs in the HR-NICE group decreased, and the proliferation and migration ability decreased as well. In terms of vascular endothelial injury and repair ability, basic diseases such as hypertension, diabetes, and hyperlipidemia could lead to a decrease in the number and quality of EPCs. The intermediate link between various risk factors and ischemic stroke is EPCs. Therefore, EPCs may be a type of more direct assessment to evaluate the high risk of NICE in the early phase and to predict prognosis. It is speculated that the cause of high stroke risk in the HR-NICE group is related to the decrease in the number of EPCs and the weakening of proliferation and migration.

It is well known that underlying diseases such as hypertension, diabetes, and hyperlipidemia are the cause of atherosclerosis and are risk factors for ischemic cerebrovascular disease. Studies have shown that the abovementioned cardiovascular and cerebrovascular risk factors can lead to a decrease in the quantity and quality of EPCs [27, 28], and the number and function of EPCs directly affects the repair ability after vascular injury. Therefore, the level and function of circulating EPCs in the ischemic brain play an important role in the pathogenesis of vascular disease.

Merwick found that patients with an ABCD2 score of less than 4 had a recurrence rate of 4.1% and suggested that the ABCD2 score should be combined with other criteria to assess the risk of recurrent stroke in TIA patients [10, 29]. Although EPCs are rarely used in clinical practice, such as stem cell transplantation and endothelial progenitor cell-seeded stents [20, 30, 31], EPCs may be an indicator to predict the risk of TIA and minor stroke. Therefore, EPCs and SDF-1 may be used as new biomarkers to predict the risk of early stroke recurrence in NICE.

## Conclusions

For TIA and minor stroke patients, circulating EPCs and serum SDF-1 concentrations can be used to prognose HR-NICE. Factors that lead to high-risk NICE might be relevant to the decrease of the proliferation and migration of circulating EPCs.

## Abbreviations

ACI: Acute cerebral infarction
BMI: Body mass index
CEPCs: Circulating endothelial progenitor cells
CI: Confidence Interval
ELISA: Enzyme-linked immunosorbent assay
EPCs: Endothelial progenitor cells
FCM: Flow cytometry
HCY: Homocysteine
HR-NICE: High-risk nondisabling ischemic cerebrovascular event
LDL: Low Density Lipoprotein
NC: Normal Control
NHR-NICE: Non-high-risk nondisabling ischemic cerebrovascular events
NICE: Nondisabling ischemic cerebrovascular events
OR: Odds Ratio
SDF-1: Serum stromal cell-derived factor-1
TC: Total Cholesterol
TG: Triglyceride
TIA: Transient ischemic attack
VEGF: Vascular endothelial growth factor
